# Tennessee Medical Society

**Published:** 1882-04-20

**Authors:** 


TENNESSEE MEDICAL SOCIETY.—The Medical Society of the State of Tennessee is appointed to meet at Memphis, on the 2d Tuesday in May next.

JOSEPH PANCOAST, M. D., Emeritus Professor of Anatomy in Jefferson Medical College, Philadelphia, died at his residence, March 7th, 1882, aged 77. He died of pneumonia

DR. W. F. GREEN, of Decatur, Georgia, died on the 6th inst., of ulceration of the bowels. Dr. Green was a kind-hearted Christian gentleman, a good physician and warmly esteemed by the entire community.

